# The bench is closer to the bedside than we think: Uncovering the ethical ties between preclinical researchers in translational neuroscience and patients in clinical trials

**DOI:** 10.1371/journal.pbio.2006343

**Published:** 2018-06-06

**Authors:** Mark Yarborough, Annelien Bredenoord, Flavio D’Abramo, Nanette C. Joyce, Jonathan Kimmelman, Ubaka Ogbogu, Emily Sena, Daniel Strech, Ulrich Dirnagl

**Affiliations:** 1 Bioethics Program, University of California Davis, Sacramento, California, United States of America; 2 Julius Centrum, Universitair Medisch Centrum Utrecht, Utrecht, The Netherlands; 3 Dahlem Research School, Freie Universitat Berlin, Berlin, Germany; 4 Max Planck Institute for the History of Science, Berlin, Germany; 5 Department of Physical Medicine and Rehabilitation, University of California Davis, Sacramento, California, United States of America; 6 Studies of Translation, Ethics, and Medicine (STREAM), Biomedical Ethics Unit, McGill University, Montreal, Canada; 7 Faculty of Law, University of Alberta, Edmonton, Canada; 8 Centre for Clinical Brain Sciences, University of Edinburgh, Edinburgh, United Kingdom; 9 Institute for Ethics, History, and Philosophy of Medicine, Medizinische Hochshule Hannover, Hannover, Germany; 10 Charité Universitätsmedizin Berlin, Berlin, Germany; 11 QUEST – Center for Transforming Biomedical Research, Berlin Institute of Health, Berlin, Germany

## Abstract

Millions of people worldwide currently suffer from serious neurological diseases and injuries for which there are few, and often no, effective treatments. The paucity of effective interventions is, no doubt, due in large part to the complexity of the disorders, as well as our currently limited understanding of their pathophysiology. The bleak picture for patients, however, is also attributable to avoidable impediments stemming from quality concerns in preclinical research that often escape detection by research regulation efforts. In our essay, we connect the dots between these concerns about the quality of preclinical research and their potential ethical impact on the patients who volunteer for early trials of interventions informed by it. We do so in hopes that a greater appreciation among preclinical researchers of these serious ethical consequences can lead to a greater commitment within the research community to adopt widely available tools and measures that can help to improve the quality of research.

For those who have the misfortune of suffering a stroke or being diagnosed with a progressive neurodegenerative disease, there are few, if any, treatments for them that will either retard or reverse symptoms, prevent major disability, or extend life. However, some will qualify for early trials testing novel drugs or biologics, representing what many see as a welcome option. Whether they realize it or not, those who enroll in these early trials will be trusting a long line of research and countless investigators whose preclinical work will have laid the foundation for the trial.

Unfortunately, the prospects for success for such trials are exceedingly low. For example, although more than 60 molecules have been investigated in the 22 years since Riluzole received marketing authorization from the United States Food and Drug Administration (FDA) for treatment of amyotrophic lateral sclerosis (ALS), there has been only one new FDA-approved drug, edaravone, as a result of all these trials [1,2]. In the case of Alzheimer disease, although clinical trials have been conducted for decades, there remains no approved drug that effectively combats the disease, as the most recent report of a failed phase III trial sadly reminds us [3]. As for stroke, despite the numerous neuroprotective drugs that ameliorate the consequences of a stroke in preclinical models, none of these drugs has been effective in patients [4].

This high rate of failure undoubtedly reflects the complexity of neurological diseases and injuries and the current limits of our understanding of their pathophysiology [5]. Further adding to the scientific challenges is the fact that few animal models mimic complex human brain phenomena, including human-type cognition, emotion, and behavior [6]. And, given their high moral status, the nonhuman primates who do share these traits are generally not available for study, either at all or in sufficient numbers.

Ethical challenges with the design of clinical trials themselves create additional hurdles that can impede progress. There are often safety concerns associated with novel interventions, such as the use of genetically modified stem cells, so phase I trials are often initially conducted on the sickest people with disorders like ALS that cause short life expectancies. This means the opportunity is lost to look for and learn about delayed safety and efficacy issues that may arise long after transplantation, information that can prove critical in subsequent initial trials in other diseases that have longer life expectancies. In addition, since many neurological disorders are disorders of suffering—e.g., severe depression, neuropathic pain—their very nature creates ethical challenges for both research ethics committees (RECs) and participant recruitment. Other degenerative disorders similarly prove ethically complex to investigate because they necessitate intervention in prodromal stages that expose “healthy at-risk” individuals to unproven and possibly unsafe treatments. Further, such studies must be of long duration, proving costly to industry sponsors.

These challenges notwithstanding, and despite the dedication of researchers, multiple, ubiquitous, and, most importantly, avoidable impediments further hinder the progress sought by all concerned. (See Fig 1) Impediments stem from a broad range of features of preclinical research that can cause problems for virtually all early clinical trials. These include, but are not limited to, matters such as low internal, construct, and external validity; exceedingly low sample sizes; nonvalidated antibodies and biologicals; and substantial publication bias. Space does not permit us to review all of these threats to the validity of the results of preclinical translational research, but meta-research of the last decade has exposed them in great detail [7–17]. To illustrate their magnitude and subsequent potential impact on the patients who enroll in early clinical trials, we will look first at matters related to publication bias.

**Fig 1:**
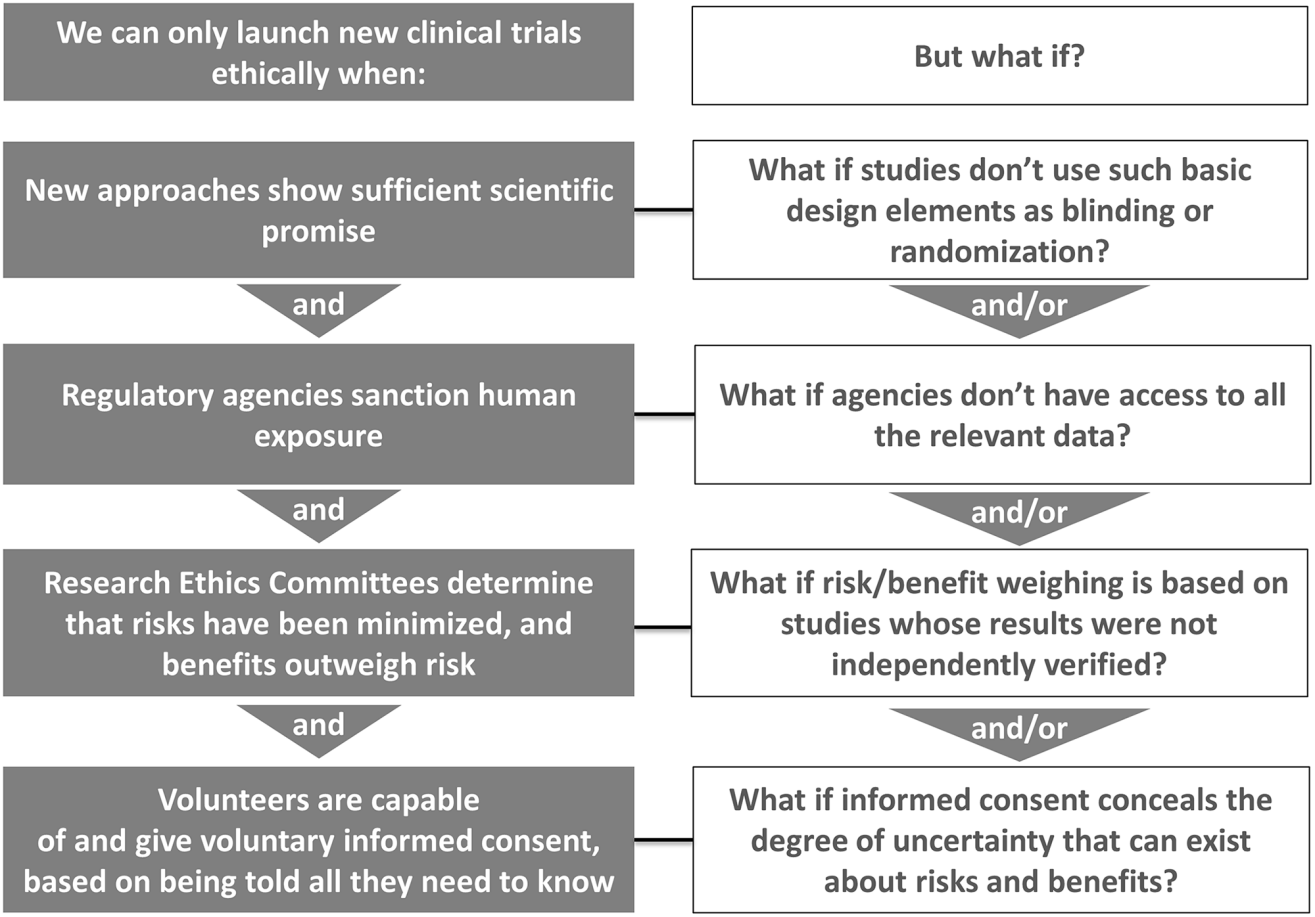
Avoidable deficiencies in preclinical research cause detrimental ripple effects all along the translation pathway that erode both the safety and ethics of early clinical trials.

If we are going to use data from preclinical studies to inform clinical trials, then the available data that describe how effective an intervention is for a given disease need to reflect adequately the entirety of data that exist testing such an assertion. This requires the publication of all experiments and outcomes assessed, irrespective of their findings. Unfortunately, experiments that find a positive effect are substantially more likely to be published than similar experiments testing the same intervention that find it not to be effective. In addition, studies that assess multiple outcomes often only report the outcomes that show a positive effect.

The bias that can result from such selective reporting is apparent in an assessment of animal studies describing neurological diseases. It observed an excess of significant findings compared to what was expected, suggesting reporting biases in the literature [18]. In the preclinical stroke literature, conservative estimates of the magnitude of the impact of publication bias have been made, and they suggest one in six experiments remain unpublished. This leads to an overestimation of treatment effects of about 30% [19]. Such studies show the extent to which current overrepresentation of positive studies—as well as the low statistical power, or “winner’s curse,” of neuroscience studies that reduces the chance that a statistically significant result is indicative of a true effect [20]—can erroneously lead us to deem an intervention to be substantially more effective than it is.

Such publication bias and the problems it poses for patients in early trials would be less prevalent if more preclinical researchers would follow the many recommendations that are available to improve the design and conduct of in vivo animal experiments [21]. Evidence from just one example of thoughtful recommendations, the Animal Research: Reporting of In Vivo Experiments (ARRIVE) guidelines, is illustrative. Developed in 2010 to improve reporting about animal research, they are now endorsed by more than 1,000 journals. The most current reports about their use show that the preclinical research community remains both largely unaware of them and recalcitrant in its uptake of them [22].

Experience with expert guidance from the Stroke Treatment Academic Industry Roundtable (STAIR) is equally troubling because it shows that it is not just individual research teams that are ignoring useful recommendations that could strengthen early trials. Federal drug approval agencies do as well. Among other things, STAIR publishes and updates recommendations for preclinical standards in the development of drugs for acute ischemic stroke [23,24]. These include expert guidance for clinical trialists on preclinical evidence requirements for launching trials. However, the corresponding European Medicines Agency (EMA) guideline for planning stroke trials does not refer to any STAIR preclinical recommendations [25], and the FDA does not provide a stroke-specific guideline.

The cumulative weight of the foregoing considerations shows that patients can enter early trials based on preclinical studies that may not have been sufficiently powered, whose investigators may not have been blinded, and the results of which may never have been replicated. One might hope that regulatory review processes would winnow out such problematic research, but the evidence on this front as well is far from encouraging. To begin with, in the US, applications to the FDA to launch initial human studies can be approved exclusively on the basis of preclinical safety data, not evidence of efficacy, revealing a narrow focus [26,27].

RECs have a broader focus, since they must make a positive determination that the potential benefits of a study outweigh its risks. They rely heavily on investigator brochures (IBs) to help them weigh risks against benefits. A recently completed study about the information from preclinical efficacy studies (PCESs) produced discouraging results [28]. It reviewed the nonclinical sections of 109 IBs for phase I/II trials submitted to German RECs over a period of six years (2010–2015). It found that reporting on PCESs infrequently describes study elements essential for evaluating those studies, including sample size (26%), baseline characterization of animals (18%), randomization (4%), sample size calculation (0%), and blinded outcome assessment (0%). For 81% of all IBs, no included PCESs had a reference to published reports. In 82% of all IBs, preclinical efficacy studies were exclusively positive. The study authors concluded that most IBs for phase I/II studies do not allow RECs—nor others such as federal regulators, investigators, or data and safety monitoring boards, for that matter—to systematically appraise the strength of the supporting preclinical findings.

Collectively, the foregoing considerations about preclinical research raise substantive concerns about whether early trials actually meet the ethical threshold found in all international codes of research ethics. Those codes stipulate that risks must be minimized and that risks must be outweighed by anticipated benefits. Equally critical is a minimum threshold for anticipated social value of a given trial [29,30]. RECs by necessity must draw upon preclinical safety and efficacy evidence in their assessment of the risks, benefits, and anticipated social value of early trials. Given the embedded problems in preclinical evidence of the sorts we have highlighted, two conclusions are unavoidable. First, the reliability of RECs’ assessments is questionable, given the documented weaknesses of the evidence they draw upon. Second, it is clear that trial participants are exposed to much more uncertainty about risks, benefits, and social value than they should be.

There is, of course, one other important ethics safeguard besides REC review that we can look to that is meant to stand as a buffer between early studies that receive REC approval and the people with serious neurological diseases and injuries who are candidates for those studies, and that is the informed consent process. But available evidence about informed consent also raises major questions. (See Fig 2) While consent documents are required to quantify risks, information about benefits is typically tied to the portion of informed consent forms explaining the purpose(s) of the study. Consequently, while forms state study objectives—i.e., what investigators hope to learn during the course of the trial—and disclose the fact that these objectives may not occur, there is no mention of how much uncertainty there is regarding whether a trial might result in the expected benefits and risks. For example, it is almost a certainty that no information is ever disclosed to potential volunteers about whether the strength of the scientific evidence relied upon to launch a trial meets basic standards of reliability, such as whether critical studies were adequately powered, whether investigators were blinded in preclinical studies, or whether regulatory approval agencies examined any efficacy data.

**Fig 2:**
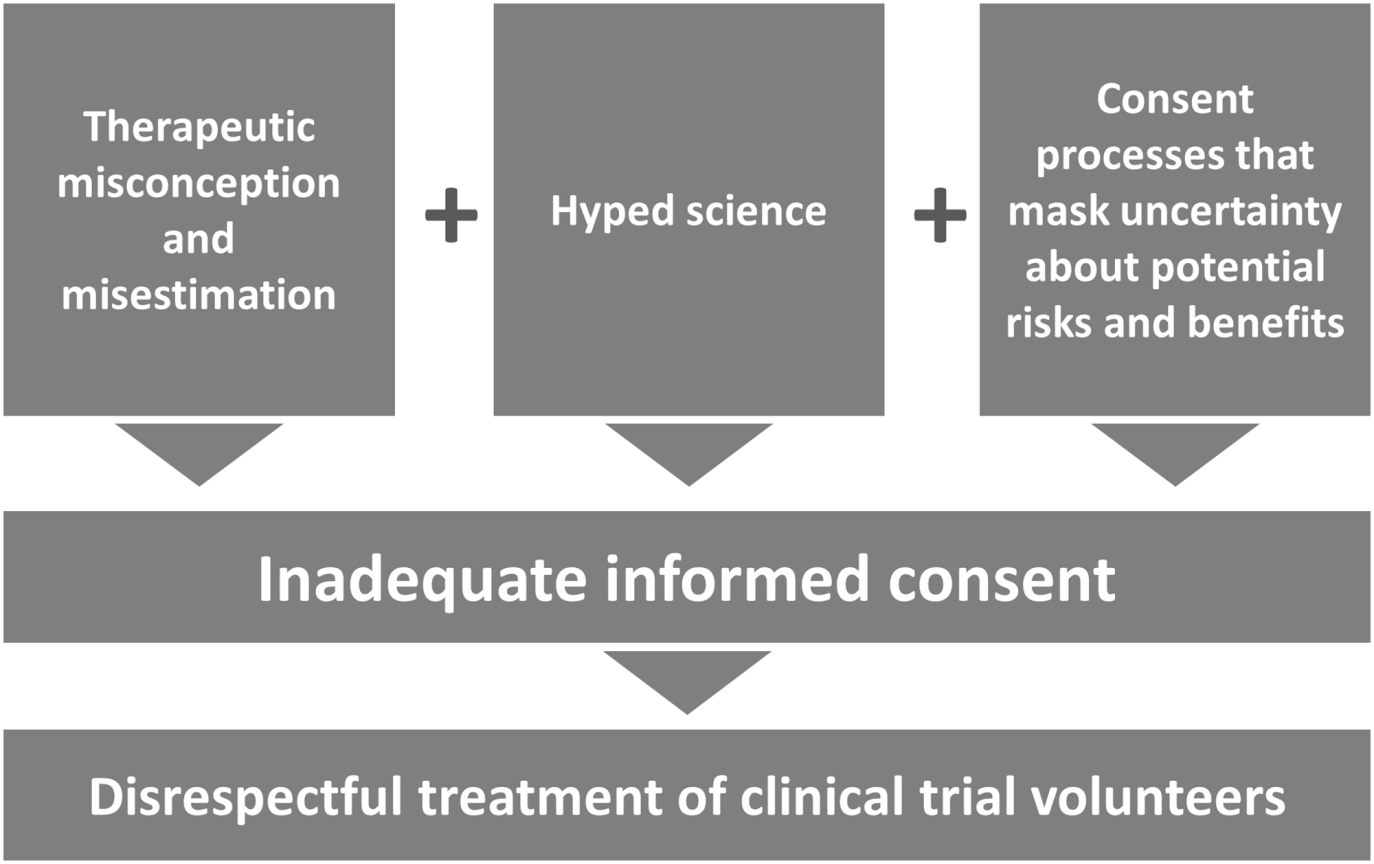
Ethically sound informed consent requires disclosure of complete and accurate information about the potential risks and benefits of early trials. Methodologically deficient preclinical studies preclude such adequate disclosures. This only compounds other well-documented problems in the informed consent process, resulting in potentially misinformed research participants.

Thus, the informed consent process will do little, if anything, to counter patient expectations that a trial is built on solid science. Nor will it offset the well-documented tendency of research participants to misunderstand critical aspects of what it means to be in a clinical trial. Research shows that participants are likely both to misunderstand how the clinical trial will differ from their regular clinical care, what is known as therapeutic misconception (TM), and to overestimate the potential benefits of participating in the trial, what is known as therapeutic misestimation (TME). Both undermine the effectiveness of informed consent for clinical trials in general and early trials of novel modalities in particular [31–33].

Informed consent processes are further weakened by well-documented problems with exaggerated portrayals of, or hype regarding, biomedical research [34,35]. This hype not only reaches participants through popular media discourse around innovative research; it also influences the discourse about research within the scientific community itself [36,37]. Hype can positively dispose clinical investigators toward trial launch and can cause trial participants to have unrealistic expectations about their trial as well, as the evidence about both TM and TME attests. Thus, it is quite likely that most volunteers enter early trials without appreciating the extent to which they are running the risk that they might make themselves even worse off than they already are.

The landscape of preclinical research and the clinical trials it supports that we have just described is the reality faced by those with serious neurological diseases and injuries who may wish to enter early trials. Their suffering is compounded by the bleak prospects that we described for breakthrough treatments that might lessen their burdens. We know that many features of their reality will not be changing anytime soon. First, there is little that can be done about the ethical complexities intrinsic to the design and conduct of early trials involving people from the affected populations. Second, regulatory bodies will be slow to change, as current efforts that began in 2011 to make changes to federal regulations governing human subject research in the US attest. This means that RECs will continue to exercise broad and, at times, flawed discretion over the trials they review [38]. And it further means that the informed consent process will continue to mask the uncertainty pertaining to the potential for both risks and benefits in early trials, since what information gets disclosed during the process is largely determined by the requirements set forth by research regulations.

Some features of the landscape of translational neurosciences that we have described are subject to change, but only if the research community musters the requisite willingness [39]. Multiple groups have long focused on matters that erode the quality and reliability of research, and they have promulgated several remedies to help address them [13,40,41]. These include measures to reduce bias and increase statistical conclusion validity [40,42–46], enforcing adherence to guidelines and recommendations [21], transparent reporting [47], and discriminating between exploratory and confirmatory research [48], among others. Adopting these kinds of reforms can have a positive impact. For example, some recent studies [49,50] have indicated that reporting of preclinical studies can be improved when journals adapt their instructions to authors.

How much of a difference widespread uptake of them would make remains unclear. Definitive evidence is lacking that robust, reliable, and reproducible in vivo modeling can, in fact, improve the prediction of success in subsequent clinical trials and the protection of patients against harm. It has to be noted, however, that the current model of drug development, as well as its regulatory framework, is based on the assumption that preclinical research regularly meets critical quality thresholds. Conversely, regardless of the model, research lacking rigor and reporting results selectively is not fit to either efficiently develop novel therapeutic strategies or assist RECs to weigh harms and benefits for patients in a meaningful way.

That is why the limited uptake of proposed remedies to improve the robustness of preclinical research is so troubling. It perpetuates many of the real-life consequences described above for the patients who volunteer for early trials. If, on the other hand, there were more uptake of them, the picture presented in Fig 1 could be significantly altered because most of the vulnerabilities of the translational process it identifies could at least be mitigated, if not eliminated. That would mean that patients could have greater trust that regulatory approval authorities and RECs could consistently draw upon strong evidence when they review and approve trials investigating new drugs and devices. As a result, the negative downstream consequences that problematic preclinical research presently bestows on patients could be lessened. That would mean that the current landscape we have described throughout this essay could be a bit brighter and the path forward in it a bit clearer.

## Abbreviations

ALS: amyotrophic lateral sclerosis
ARRIVE: Animal Research: Reporting of In Vivo Experiments
EMA: European Medicines Agency
FDA: Food and Drug Administration
IB: investigator brochure
PCES: preclinical efficacy study
REC: research ethics committee
STAIR: Stroke Treatment Academic Industry Roundtable
TM: therapeutic misconception
TME: therapeutic misestimation
